# Movement beyond data: epistemic and pictorial challenges in understanding moving life

**DOI:** 10.3389/fbinf.2026.1762759

**Published:** 2026-02-19

**Authors:** Janina Wellmann

**Affiliations:** 1 RG Premodern Sciences, Max Planck Institute for the History of Science, Berlin, Germany; 2 FG Wissenschaftsgeschichte, Technische Universitat Berlin, Berlin, Germany

**Keywords:** molecular motion, molecular motors, morphogenesis, movement perception, visualization

## Abstract

Nothing inside organisms is at rest; everything moves. Cells are transported through the vascular system, proteins move cargo away or toward the cell nucleus, and enzymes repair DNA, which is constantly modified by metabolic cell processes or external influences affecting the organism. Various processes occur simultaneously in the crowded spaces of cells and across tissues, carefully coordinated and orchestrated. In contemporary science, movement lies at the root of studying cellular and molecular processes—in short, of all the activities that occur within the organism. This article provides a historical perspective and methodological reflection on the study of cellular and subcellular motion in current biotechnology. It shows that, far from being evident, movement is not simply observed but actively made. I argue, first, that the conceptualization of motion in current biotechnology occurs through image-making and is thus shaped by a long pictorial history and the struggle to depict movement. Second, to make the invisible move under the conditions of visibility, metaphors and imaginaries drawn from our everyday experience of animal motion are transposed into the nanoscopic sphere, thereby setting the framework and limits of understanding motion at the molecular level.

## Introduction: perceiving movement

When the world of microorganisms was first discovered by Antoni van Leeuwenhoek (1632–1723) at the end of the 17th century, he was thrilled by the agility of the microorganisms he had discovered under his lens, which no one had seen before him. If the tiny animals were a mystery, their motion was even more so. In great detail, Leeuwenhoek describes the various movements of the beings he identified as organisms taken from the water of the well in his yard, the sea, or infusions of ginger, nutmeg, and pepper (Leeuwenhoek, 1677; Leeuwenhoek, 1684a). The animals move by extension and contraction, thin feet, straightening, bending, or jumping; they resemble gnats in the air, eels in water, or fleas and serpents on the ground; and their motion is characterized as nimble and brisk, gentle and swift, or pretty and pleasing (Leeuwenhoek, 1677, 821–831; Dobell, 1932, 140–143, 149).

When Leeuwenhoek writes of “living creatures,” “animalcula,” and “living atoms”—often using the Dutch diminutives *dierken* or *kleine dierkens*—he means very small animals, whether they can be observed with the naked eye or only through the microscope (Leeuwenhoek, 1677, 821, 827). At the time of Leeuwenhoek’s discovery, little could be said about life except that it moves. The sole reason why Leeuwenhoek is so sure he is seeing animals, then, is that they are moving. The forms of that movement, deciphered with care and admiration, are no mere curiosity, but the very proof of their identity (Dobell, 1932, 201). What moves is an animal—on both sides of the lens, regardless of its size. The question of what exactly he had seen through the lens, then, was a matter of magnitude only: the transition from the world this side of the lens to the world on the other side was seamless (Leeuwenhoek, 1677, 821–827).

While Leeuwenhoek was the first to perceive and pay attention to microscopic movement, morphogenesis was one of the earliest fields in biology where movement was found crucial in driving generation and shape changes during development *inside* organisms. Embryology became a scientific discipline around 1800 (Gilbert, 1991; Roe, 1981; Roger, 1963; Smith, 2006). The major discovery at the onset of the investigation of chick embryos was the finding that all the shapes of the developing embryo are brought about by the folding of the germ layers (Pander, 1817; Baer, 1967 [1828/1835]). The fold produces a form along its line; folding is the choreography of space–time movements of the embryonic membranes: every warp, swelling, or indentation, at every location and at every moment, directly changes the space–time coordinates of the entire embryo (Wellmann, 2017). A hundred and 50 years later, at the end of the 19th century, cell movements were found to be at the core of gastrulation, the process early in embryo formation that brings inside the embryo cells that lie further outside in order to form the germ layers.

As a phenomenon manifesting itself in continual change and momentary consummation, it is performance and reiteration that give shape to movement. Movement is not an object to be found; it happens. Evading perception and representation alike, it is hard to portray. As an event, it has a perceptual, cognitive, and participatory dimension and thus occurs within a situated interplay of place, time, and viewer. When it comes to conceptualizing motion, we have to examine how it is produced—both as an event and in technologies of reproduction. Not surprisingly, art, technology, and the mind have been challenged by the question of how to capture, freeze, and extract motion from flux in a concerted interaction of perceiving, conceiving, and producing motion (Wellmann, 2024).

## Making movement

**FIGURE 1:**
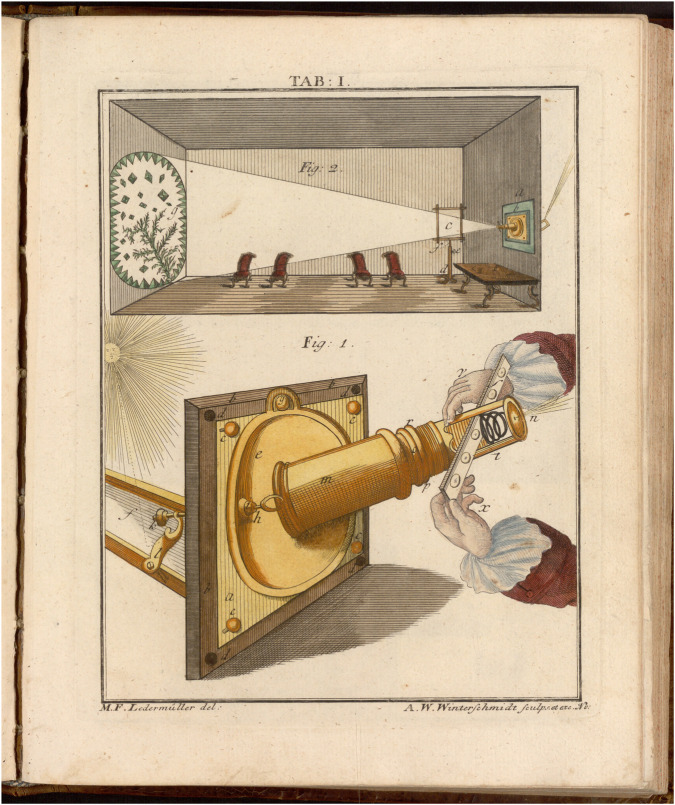
The solar microscope projects a magnified object onto the wall of a darkened room in (Ledermüller, 1762, plate 1).

Leeuwenhoek vividly described in words the movements of his newfound animals. However, in his pictures, they were immobilized. His depictions with pen and pencil failed to make his readers participate in seeing what he himself observed. His imagery of the marvel of blood’s circulation, the beautiful rotational movement of the wheel-like rotifer, or an animalcule darting through space, does not remotely reflect his sensual experience (Leeuwenhoek, 1702; Leeuwenhoek, 1703; Leeuwenhoek, 1684b). A static depiction of motion is not, and cannot be, motion itself. Already in Leeuwenhoek’s day, however, technologies existed that allowed the observer to produce motion as an event. Projection with the camera obscura or magic lantern, using light and lenses, offered a space of experimentation for a new, sensual experience of microscopic life as it unfolded before one’s eyes (Hammond, 1981; Hecht, 1993). Not least, Leeuwenhoek himself, secretive as he was about the technology he created, is likely to have experimented with light and projection to enlarge his observation of minuscule organisms and their behavior (Huerta, 2003; Snyder, 2015, 296–98; Staedman, 2001, 44-58). In the 18th century, solar microscopy made it possible to cast living motion ceiling-high onto the wall of a darkened room to the delight of an enlightened audience (Figure 1) (Heering, 2008; Wellmann, 2008). Transformed from the world of the microscopically small, invisible to the human eye, into the world of the spectator, living movement could now be grasped with all senses. Moreover, the solar microscope made accessible movements so slow or delicate that they became only perceptible when enlarged and projected. In that capacity, the solar microscope was still in high regard in the 19th century, for example, at the Collège de France, where François Magendie held the first chair of experimental physiology (La Berge, 1994). What could be observed only through the lens, in the ephemeral play of light and shade, however, has largely been lost and has not found its way into our knowledge of 17th and 18th century microscopic explorations. Not only were the pictorial means missing to capture the fugitive nature of the projections and the motion they displayed, but being performative, such projections were disregarded as a transient spectacle, an entertainment—designed to frighten or amuse the uninitiated, untouched by scientists, and enjoyed by amateurs only within the constraints of a gentlemanly practice (Mannoni, 2000; Gunning, 2004, 31–44; Stafford, 1994).

Causing no less unease is the fact that, for most of history, the living qualities of organisms were studied with the help of organisms that were not alive but dead. Well into the 20th century, the living world had been stilled by the instruments needed to get access to it (Fox Keller, 2002, 218). Under the microscope, organisms were stained, fixed, and cut into slices; extracted from their environments; mounted; and fragmented. In morphogenesis, making sense of cells in motion, thus, presented scientists with a substantial challenge. Working with dead substances, movement could not be seen. How, then, could it be understood? Conversely, if cells were studied in the living specimen and hence visible but fleeting, how could movement be captured, traced, or archived? Once cells were seen and investigated as moving actors, a new and major problem immediately arose: How do cellular movements bring about the shapes of the developing organism? How should one understand the emergence of form out of hundreds of diverse, delicate, erratic movements?

In the first decades of the 19th century, the study of cell motion in development started with rulers and measuring distances between cells in histological preparations and drawings. The method was soon complemented by serial photography, but it was the advent of cinematography in the 1920s that introduced a new dimension into the scientific interrogation of movement inside organisms. To the scientific pioneers, film allowed an “intimate glimpse” into the “morphology and physiology of development” (Gräper, 1926, 55) and invited the spectator to “truly ‘take part’ in the events” (Gräper, 1929, 383). Studying form by aggregating information from many specimens, sections, or snapshots was a statistical approach, which they believed did not contribute to understanding movement and explaining morphogenesis. Promising to render cell movements visible in a single specimen over time, film seemed to be the prerequisite for the scientific analysis and explanation of the phenomenon of movement. Only with film as a descriptive device to trace movements did the phenomenon they wanted to examine begin to exist. As it turned out, however, film did not allow clarifying the core question of early cell movements in avian gastrulation, i.e., how to understand the three-dimensional flows by which the cells were exchanged between the surface and the interior of the ovum to form the two primitive streaks. After years of labor on experimental set-ups, imaging devices, and technological refinement, the German embryologist Ludwig Gräper of Leipzig invented a stereocinematographic apparatus (Michaelis, 1955, 58). The new approach finally allowed him to conceptualize the complex time–space choreography of cell movement in gastrulation: the cells were dancing. More precisely, he compared their choreographed movements to an 18th century ballroom dance, a polonaise (Gräper, 1929, 391, 401).

Cast aside as merely descriptive, though, film faced strong opposition from the beginning. Starting around 1900, developmental mechanics and a new experimental approach were guided by an interest in geometry and the search for general physical principles in organ formation. To apply mechanistic principles meant to frame the problem of emerging form as one of the deformation of shape. In 1902, the zoologist Ludwig Rhumbler, for example, embarked on tactile modeling of shape changes by building physical models, in which he combined different materials (wire and corset slats) to mimic various mechanical properties of the organic structures in question, here the blastula and its inward curving or invagination of the original spherical shape (Rhumbler, 1902). It took almost a century until computation introduced new ways of modeling and simulating the same problem of invagination that Rhumbler had tackled physically with wires and steel slats. In the 1980s, G. M. Odell developed one of the first computer models of gastrulation. Based on a mechanical model of a cell, the “shape history” of each cell is computed by solving a set of differential equations (Odell et al., 1981, 448–449, equations on p. 457). Subsequently, the “dynamical behavior”’ of assemblies of cells is examined by varying the geometrical and mechanical parameters, making it possible to simulate the behavior of the cell shape changes over a certain time period (Odell et al., 1981, 449). The approach was deemed superior to descriptive recording on film, as computational modeling, instead, allowed for an explanation of the forces driving movement (Odell et al., 1981, 446). At the end of the century, increasing computer power and finite-element simulations pushed the geometrical framing of shape change in morphogenesis another step further (Davidson et al., 1995).

In short, understanding the dynamics inside organisms from the point of view of cell motion or shape change yielded entirely different perceptions, methodological approaches, technological tools, and visual imaginaries, to the point of being mutually exclusive. However, modelers and simulators regarded cinematographers’ work as merely descriptive; cinematographers, in turn, considered the modelers’ work impotent, offering merely aggregated, statistical, and possible scenarios without elucidating the actual situation in an individual ovum.

## Vocabularies of movement

As we have observed, the transition from the macroscopic to the microscopic was seamless in the 17th century. Tiny creatures on the other side of the lens were animals by virtue of their movement; therefore, they had to swim, tumble, or fly like their counterparts in everyday perception. Tracing cell movement in the three-dimensions of space brought to the fore the enormous complexity of elaborate, orderly, highly coordinated movements of cell groups. To make these movements intelligible, they first had to be made visible and then were captured in the language of dance, which provided the most detailed framework to describe the spatio-temporal choreography of various actors moving individually and yet as members of a larger whole.

**FIGURE 2:**
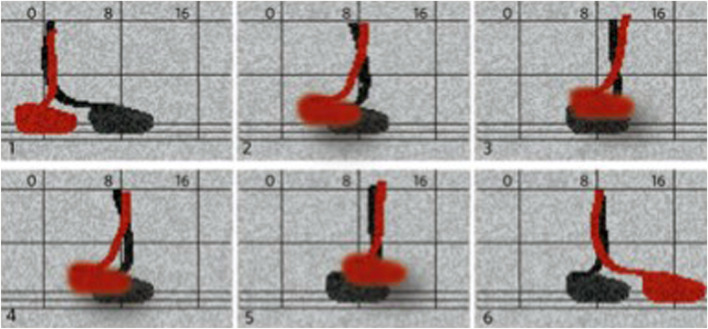
Cartoon depiction of the gait of the motor protein kinesin in (Peterman, 2016, Figure 1).

That vocabulary of animal and human motion in the subcellular sphere is not lost in twenty-first-century biotechnology’s words and visuals; it is quite the contrary. The post-genomic life sciences have found a new vitality due to live-cell imaging technologies. Biology has become a science of structures in motion since these technologies make it possible to observe processes as they occur in living cells by labeling cells and subcellular elements with fluorescent markers, capturing their behavior with high-resolution microscopes, and processing the resulting data with computer technology. In 2005, applying live cell imaging and vector analysis to gastrulation, time-lapse sequences, and tracking algorithms “confirmed that, during streak formation, cells flow from the posterior sickle toward the posterior midline…in a polonaise movement” (Cui et al., 2005, 38). At the level of molecular motors, to provide another example, walking kinesin has made the headlines. Actin, myosin, and kinesin are proteins. Their biological potential unfolds through their distinctive combinatorics of composition, spatial structure, and activity. Chemically, they are enzymes, ATPases that, as biological catalysts, are involved in the process of splitting ATP into ADP. While this chemical reaction, dephosphorylation, releases energy, structurally, it results in the protein’s shape change, or conformational change. Kinesin is termed a motor, then, because the conversion of chemical energy into mechanical motion is what motors are designed for. Although motors rarely walk, it is the gait of kinesin that troubled the scientists. Relying on the exact correspondence of biochemical and mechanical processes, the engineering model makes kinesin, which structurally consists of two “heads,” perform “power strokes,” “step,” and “walk” on the microtubule, which serves the protein as a substrate (Figure 2) (Howard et al., 1989, 154). However, the prevalent asymmetric hand-over-hand model of kinesin stepping, does not entirely resolve the issue of how chemical and mechanical steps are coupled. The asymmetry of the model raises the question of whether kinesin does not actually walk but “limps” (Schliwa, 2003).

## Conclusion

Whether with the naked eye, bead lenses, or nanoscopy, by way of the hand, film, or modeling, and despite all technological advances, the imaginary of motion remains closely linked to its 17th century origins. Since Leeuwenhoek seamlessly moved between the spaces of what was only just visible and what just needed optical enhancement, movement in the biological world and deep down into nanoscopic invisibility is framed by our experience and knowledge of the multifarious ways in which animals move in the world we share. This holds true despite the fact that the laws of macroscopic physics do not apply to the molecular level. Contemporary simulation, live-cell imaging, computer animations, and AI, however, not only render intelligible data in a world of research that can no longer think, calculate, or experiment without moving images. They also continue to inscribe the living world into the cultural register of movies. A modern form of enchantment, they transform the spectator’s experience of the internal obscurity of the organism into a spectacle of actors being illuminated and rendered at will by technology: here, the motor protein kinesin becomes a nimble sapling walking on a self-assembling track, the microtubule as in the 2007 Harvard animation “The Inner Life of the Cell” (Wellmann, 2024, 190ff).

The motion of living beings and inside of them is the living world’s inherent promise and potential for a future. What is new in contemporary life sciences is that molecular motion lies at the heart of mechanism. Looking at how biological motion has been made and interpreted in the past, then, informs us not only about the past but also the present day. With biotechnology and synthetic biology advancing toward the creation of artificial life, or parts thereof, they must take ownership of this promise for the future. For the time being, biological motion sets limits to what biology can do. Life is movement, and a science of life rests on knowledge of how we make that movement. The concrete manner by which movement is generated is the key to understanding it.

## Data Availability

The original contributions presented in the study are included in the article/supplementary material; further inquiries can be directed to the corresponding author.
